# The alfalfa AP2/ERF transcription factor *MsCBF4* enhances frost tolerance in *Arabidopsis thaliana*

**DOI:** 10.1186/s12870-025-07778-y

**Published:** 2025-11-24

**Authors:** Nai-Peng Ren, Hui Zang, Yi-Fu Xuan, Jie-Lin Liu, Xiang-Ping Liu, Guo-Liang Li

**Affiliations:** 1 College of Agriculture, Heilongjiang Bayi Agricultural University, Daqing, 163319 China; 2 College of Animal Science and Veterinary Medicine, Heilongjiang Bayi Agricultural University, Daqing, 163319 China; 3 Grassland Research Institute of Heilongjiang Academy of Agricultural Sciences, Harbin, 150086 China

**Keywords:** Alfalfa, MsCBF4, Cold stress, Freezing stress, Antioxidant system, Arabidopsis thaliana

## Abstract

**Background:**

Alfalfa (*Medicago sativa* L.) is globally important forage crop whose survival and productivity at higher latitudes depend on cold-stress tolerance. However, the molecular mechanisms of how alfalfa defends against freezing stress remain unclear.

**Results:**

In this study, the candidate gene *MsCBF4* was cloned from the cold-tolerant alfalfa genotype “Dongnong NO.1”. *MsCBF4* belongs to the APETALA2/Ethylene Response Factor (AP2/ERF) transcription factor family and functions as a transcriptional activator via a C-terminal activation domain. The encoded protein localized to the nucleus. *MsCBF4* is induced by cold and freezing conditions and is expressed in roots, stems, leaves, flowers, and pods, with the highest expression levels in roots and leaves. Overexpression of *MsCBF4* in *Arabidopsis thaliana* seedlings significantly enhanced root length, lateral root number, and fresh weight under cold stress. Lines overexpressing *MsCBF4* demonstrated enhanced frost tolerance at both the seedling and mature stages, likely through improved physiological scavenging of reactive oxygen species (ROSs), thereby limiting membrane damage. Furthermore, under freezing stress, the expression levels of key genes involved in abiotic stress resistance were upregulated in transgenic *Arabidopsis*. The *MsCBF4* promoter exhibits transcriptional activity and can be directly activated by cold responses. Yeast one-hybrid (Y1H) and dual-luciferase reporter (Dual-LUC) assays showed that *MsERF6* specifically binds to the *MsCBF4* promoter and positively regulates its expression.

**Conclusion:**

*MsCBF4* enhances frost tolerance in *Arabidopsis* by alleviating oxidative stress, protecting membrane integrity, and upregulating stress-responsive genes.

**Supplementary Information:**

The online version contains supplementary material available at 10.1186/s12870-025-07778-y.

## Background

 Freezing stress is a type of abiotic stress faced by plants, occurring at temperatures below the freezing point (< 0℃). Freezing may directly damage plant tissue, leading to the dehydration of protoplasm and damage to subcellular fine structures. Such damage is often irreversible and can result in plant death [1, 2]. Alfalfa (*Medicago sativa* L.), a high-quality perennial forage crop in the legume family, is widely cultivated worldwide [3]. However, the harsh winter weather in northeastern China makes plants susceptible to freezing stress. This stress reduces crop productivity and significantly decreases the likelihood that crops will survive the winter [4]. Alfalfa has evolved a series of coping mechanisms under prolonged freezing stress, including cold acclimation processes involving signal perception and preparatory responses [5], as well as physiological, biochemical, and molecular responses [6]. Transcription factors (TFs) play a key role in mediating these gene-expression regulatory mechanisms and are important in alfalfa’s response to cold stress [7]. Members of the APETALA2/Ethylene response factor (AP2/ERF) family is crucial for increasing a plant’s ability to withstand cold [8].

The AP2/ERF family is widely involved in the biological and abiotic stress processes in plants and is one of the largest families of plant-specific transcription factors [8]. Members typically contain 1–2 highly conserved AP2 domains, consisting of 60 to 70 amino acids (AA), which form a characteristic helix-turn-helix structure and regulate downstream target-gene through specific DNA binding [9, 10]. As an important member of the AP2/ERF family, the C-repeat Binding Factor (CBF) is a core regulator of plant responses to cold stress [11]. During the cold acclimation process of *Eucalyptus globulus*, the genes *EglCBF1a*, *EglCBF1c*, and *EglCBF1d* play significant roles. Correspondingly, frost-tolerant phenotypes have been observed in transgenic *Arabidopsis thaliana* [12]. In *Malus baccata*, the overexpression of *MbCBF2* in transgenic *Arabidopsis* consequently increased the expression of the downstream genes: *AtCOR15A*, *AtRD29A/B*, *AtCOR6.6/47*, *AtCAT1*, *AtP5CS*, and *AtPIF1/4*. This enhanced tolerance to low-temperature stress [13]. Several CBF gene sequences that respond to low temperatures have been identified in *Medicago falcata* and *Medicago truncatula* [14]. Studies have also shown that nine CBF TFs that are differentially expressed are homologous to the CBF gene at the Mt-FTQTL6 locus. It has been proposed that these CBF TFs may confer frost tolerance to *Medicago truncatula* [15]. Additionally, the stress-tolerance function of CBF genes has also been reported in response to abiotic stresses, such as drought and salinity. The *MbCBF1* gene from *Malus baccata* enhances the adaptation and tolerance of transgenic *Arabidopsis* to high-salinity environments [16]. The overexpression of the *MtCBF4* gene from *Medicago truncatula* improves the tolerance of *Arabidopsis* to drought and salt stress [17]. Although CBF genes have been identified in various species in response to abiotic stress, their role in frost tolerance remains poorly understood in alfalfa.

In this study, based on RNA-Seq data from the cold-tolerant alfalfa genotype “Dongnong NO.1”, the coding sequence (CDS) and promoter region of the significantly upregulated gene *MsCBF4* were cloned. *MsCBF4* was characterized through bioinformatics analysis and gene-feature annotation. Its tissue-specific and spatiotemporal expression patterns were also examined. To assess its functional role, the performance of transgenic *Arabidopsis* expressing *MsCBF4* was evaluated under cold and freezing stress. Promoter activity was validated using β-glucuronidase (GUS) staining and transient expression. Meanwhile yeast one-hybrid (Y1H) and dual-luciferase reporter (Dual-LUC) assays revealed that the *MsERF6* protein regulates the *MsCBF4* promoter. Together, these findings provide a theoretical foundation for understanding frost tolerance in alfalfa and support the development of new cold-resistant germplasm.

## Results

### MsCBF4 cloning and sequence analysis

**Fig. 1:**
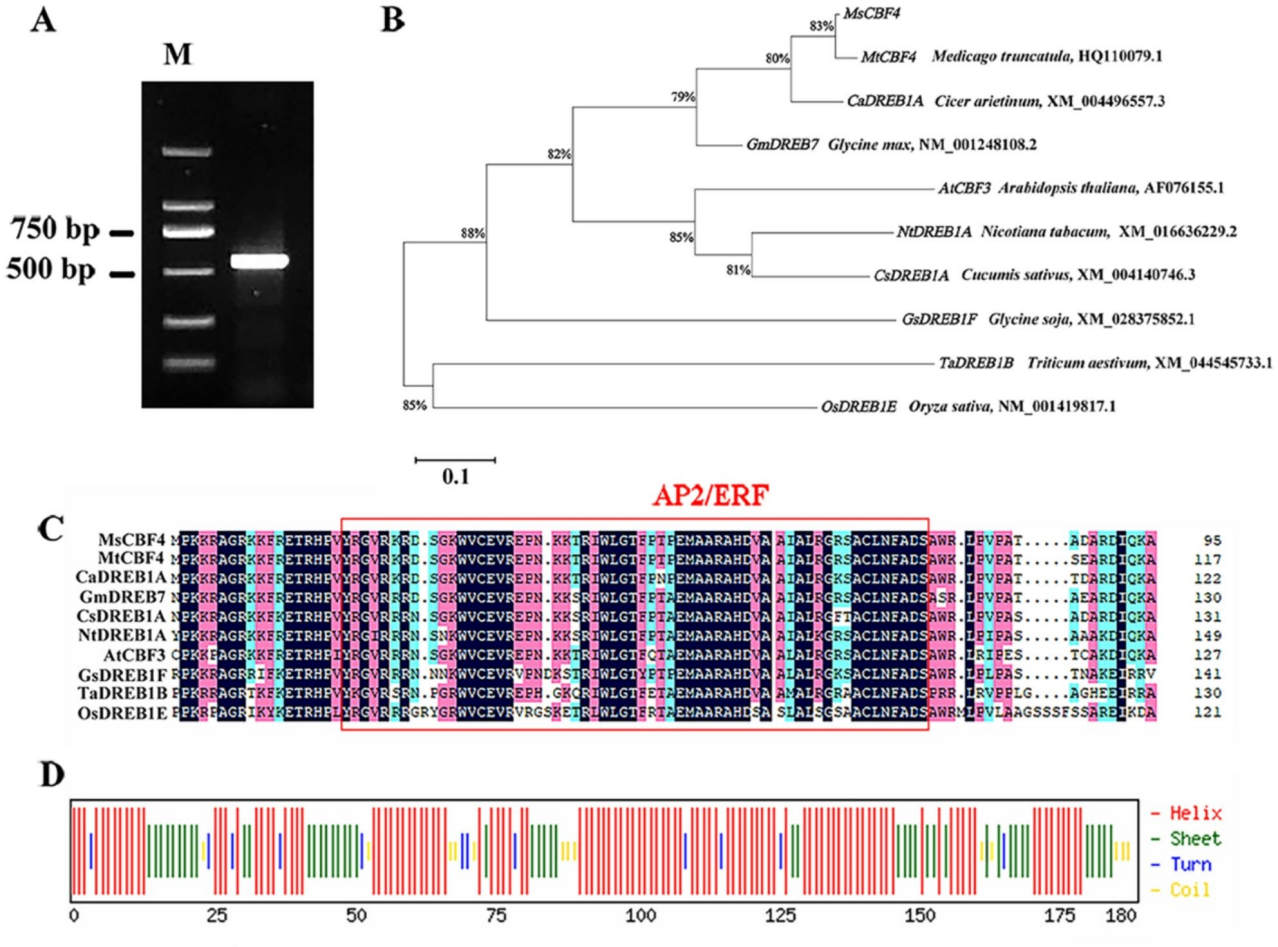
Cloning and sequence analysis of *MsCBF4*. **A** ORF sequence cloning. **B** Phylogenetic analysis. **C** AA sequence alignment of MsCBF4 with other homologous proteins. **D** Prediction of the secondary structure of the MsCBF4 protein

Based on previous transcriptomic sequencing results, we observed that the gene *MS.gene006341.t1* in the cold-tolerant “Dongnong NO.1” genotype was significantly upregulated after 2, 4, and 6 h of freezing treatment (Table S1). The corresponding alfalfa gene was annotated as *MsCBF4* using the homologous sequence of *MtCBF4* in the *Medicago truncatula* genome (Fig. 1B). The open reading frame (ORF) of *MsCBF4* was subsequently cloned from the “Dongnong NO.1”. The ORF is 543 bp in length and encodes a 180 AA protein containing a conserved AP2/ERF domain of 58 AA (Fig. 1A). The predicted molecular weight of *MsCBF4* is 20.44 kDa, with a theoretical pI of 5.76. With an instability index of 64.25, MsCBF4 is categorised as an unstable protein. Furthermore, MsCBF4 is a hydrophilic protein that lacks both transmembrane domains and signal peptides (Table S2). Phylogenetic analysis showed that *MsCBF4* exhibits a high degree of homology with *MtCBF4* from *Medicago truncatula* (Fig. 1B). Additionally, all of the protein sequences encoded by these genes contain a conserved AP2/ERF domain (Fig. 1C). Secondary-structural predictions indicate that the MsCBF4 protein consists of 60.56% α-helices, 25.56% β-sheets, 6.67% β-turns, and 7.22% random coils (Fig. 1D). Tertiary structure prediction indicates that the core domain of the MsCBF4 protein is highly conserved and similar to that of AP2 family TFs (Fig. S1).

### *MsCBF4* transcriptional activation activity and subcellular localization

**Fig. 2:**
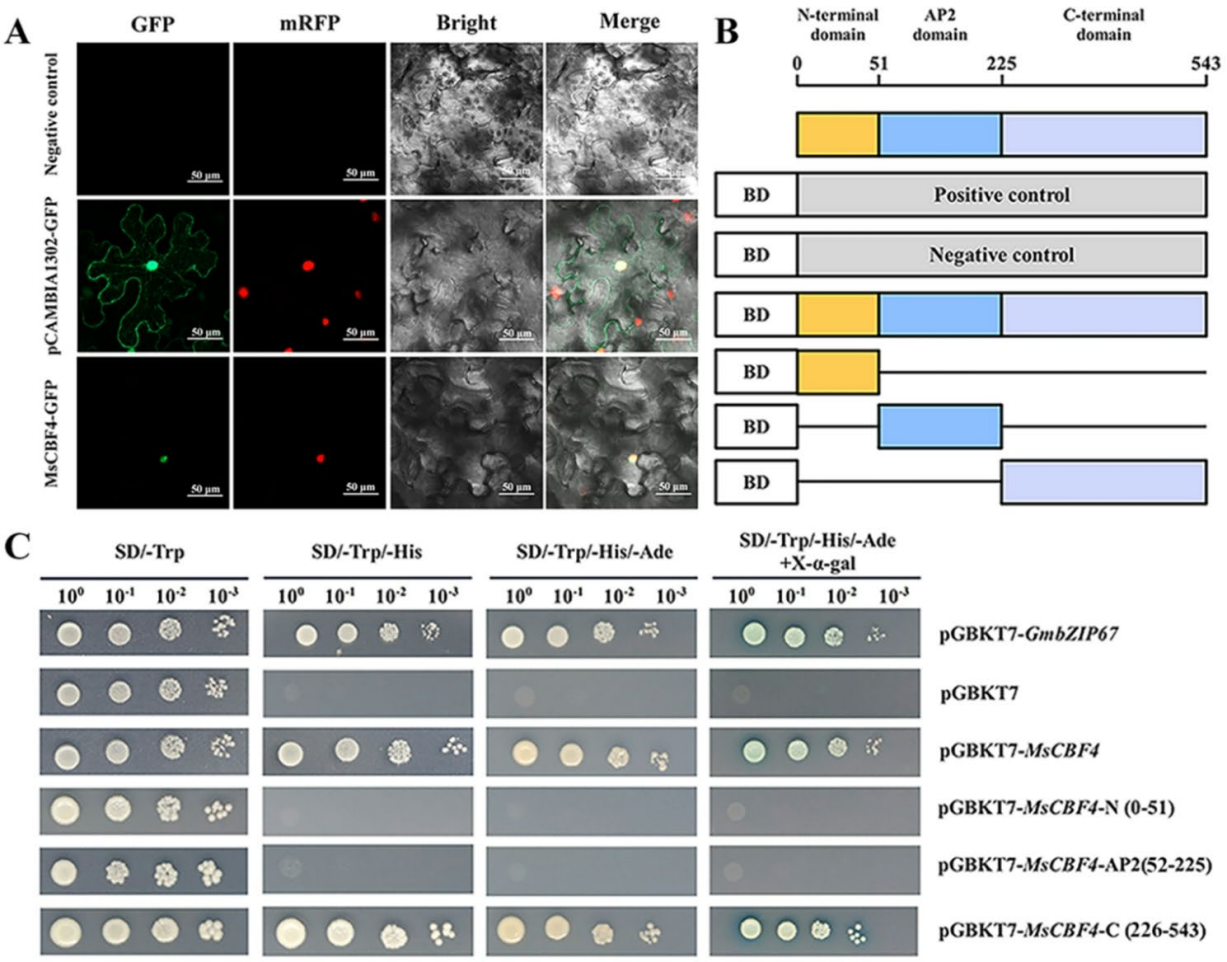
Subcellular localization and transcriptional activation analysis of MsCBF4. **A** Subcellular localization of MsCBF4. mRFP indicates the location of the cell nucleus, scale bar = 50 μm. **B** Schematic diagram of the truncated full-length sequence of pGBKT7-*MsCBF4*. **C** Transcription activation analysis of pGBKT7-*MsCBF4* in transformed Y2H yeast strains

Subcellular localization predictions indicate that the MsCBF4 protein is localizes to chloroplasts, cell nuclei, and mitochondria (Table S2). After transferring the pCAMBIA1302-*MsCBF4*: GFP into the *Agrobacterium tumefaciens* GV3101 strain, transient expression was achieved on the back of tobacco leaves. Results from fluorescence distribution analysis showed that the green fluorescence of pCAMBIA1302-GFP was localized in the cytoplasm and nucleus. In contrast, MsCBF4-GFP fluorescence was only detected in the nucleus and overlapped with the *AHL22*-mRFP marker. This provides evidence that the MsCBF4 protein is nuclear-localized (Fig. 2A). Furthermore, Y2H Gold cells transformed with pGBKT7 only grew on SD/-Trp medium. In contrast, the positive controls, pGBKT7-*GmbZIP63* and pGBKT7-*MsCBF4*, grew on all three nutrient-deficient media and turned blue on SD/-Trp/-His/-Ade medium supplemented with X-α-gal (Fig. 2C). The pGBKT7-*MsCBF4* fragment was cleaved into three segments: the N-terminal domain, the AP2 domain, and the C-terminal domain (Fig. 2B). Only the sequence corresponding to the C-terminal domain of *MsCBF4* supported normal yeast growth (Fig. 2C). These results demonstrate that *MsCBF4* acts as a transcription activator, with its transcriptional active region located to the C-terminal domain.

### MsCBF4 is strongly induced by cold and freezing stress in alfalfa

**Fig. 3:**
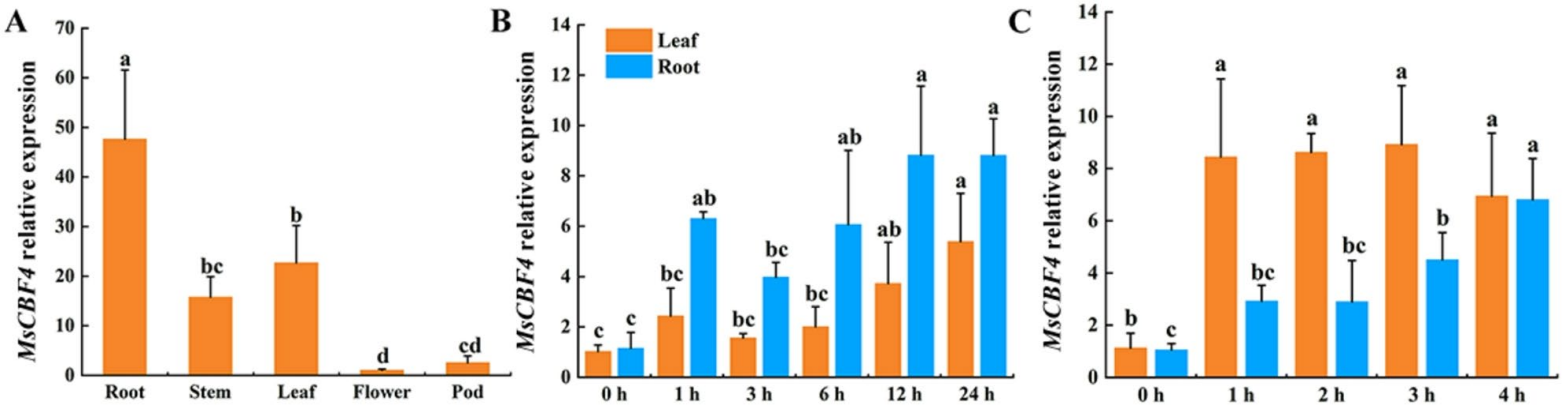
Expression profiling analysis of *MsCBF4*. **A** Expression of *MsCBF4* in roots, stems, leaves, flowers, and pods of 13-week-old alfalfa plants. **B** Expression of *MsCBF4* in roots and leaves of 5-week-old alfalfa during cold treatment (4℃). **C** Expression of *MsCBF4* in roots and leaves of 5-week-old alfalfa plants after 2 days of cold adaptation at 4 °C during freezing treatment (-5℃). Data are presented as mean ± SD (standard deviation) of three independent samples. Different letters indicate significant differences at the tissue (A) and time (B, C) levels with *P* < 0.05

The expression of *MsCBF4* in alfalfa tissues was analyzed using quantitative reverse transcription polymerase chain reaction (qRT-PCR). *MsCBF4* was found to be expressed in all tissues, with the the highest levels observed in roots and the lowest in flowers (Fig. 3A). *MsCBF4* expression peaked after 24 h of cold treatment in leaves, whereas in roots it peaked after 12 h and then declined (Fig. 3B). In leaves, *MsCBF4* expression was induced after only 1 h of freezing treatment and stabilized quickly, whereas in roots expression peaked after 4 h of freezing treatment (Fig. 3C). Overall, *MsCBF4* expression was higher in roots than in leaves during cold exposure. Conversely, it was higher in leaves than in roots during freezing treatment. These observations suggest that *MsCBF4* is induced by cold and freezing conditions, with greater expression sensitivity exhibited in roots during cold treatment and heightened sensitivity in leaves during freezing.

### MsCBF4 promoter cis-acting elements and activity analysis

**Fig. 4:**
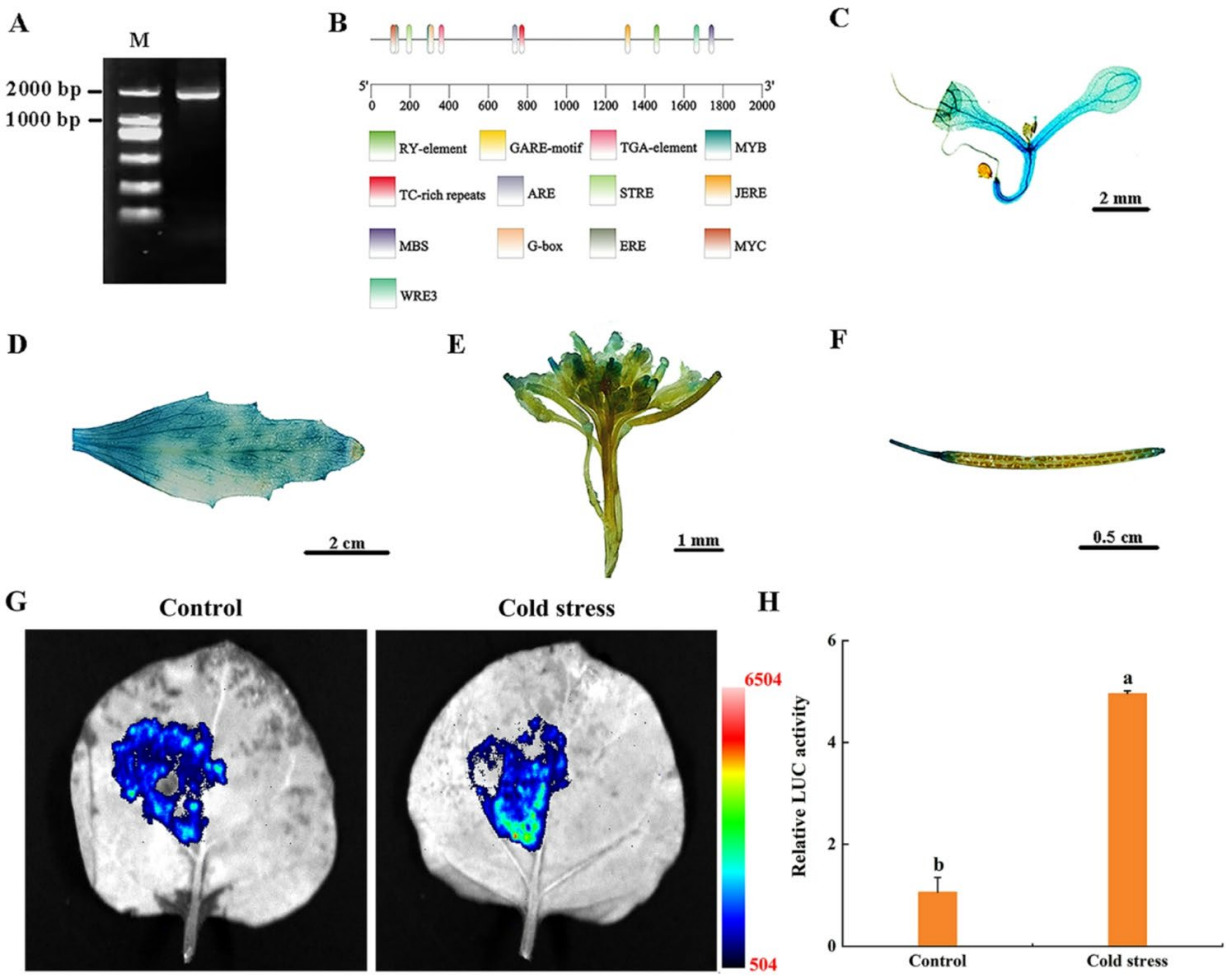
Cloning and analysis of the *MsCBF4* promoter. **A** Cloning of the promoter sequence. **B** Cis-acting elements predicted in the promoter. **C** GUS staining of transgenic *Arabidopsis* seedlings. **D** Leaf, **E** inflorescence, and **F** pod tissue GUS staining of transgenic *Arabidopsis*. **G ***ProMsCBF4* activity in tobacco leaves under at room temperature conditions and after three hours of cold stress. **H** Relative LUC activity. Data are presented as mean ± SD of three independent samples, and different letters indicate significant differences (*P* < 0.05)

To investigate the function and molecular mechanism of the *MsCBF4* promoter, a 1793 bp sequence was obtained from the upstream flanking regulatory region of the ORF from the genomic DNA of alfalfa “Dongnong NO.1” (Fig. 4A). Analysis of cis-acting elements within the *MsCBF4* promoter region identified multiple stress-responsive motifs, including MYB, STRE, JERE, ERE, ARE, G-box, and TC-rich repeats (Fig. 4B). To investigate the transcriptional activity and tissue-specific expression pattern of the *MsCBF4* promoter, a pCAMBIA3301-*MsCBF4*pro: GUS fusion vector was constructed and transformed into *Arabidopsis*. Histochemical GUS staining results demonstrated positive blue staining in transgenic *Arabidopsis* seedlings, mature leaves, inflorescences, and siliques (Fig. 4C-F). Subsequently, the *MsCBF4* promoter was cloned into the pGreen II 0800-LUC vector to generate the *MsCBF4*pro: LUC recombinant construct, which was then transiently expressed in tobacco leaves under both room temperature (control) and cold stress conditions. Relative firefly luciferase (LUC) activity was significantly higher under cold stress compared to control (Fig. 4G, H). Collectively, these findings confirm that the *MsCBF4* promoter exhibits transcriptional activity across multiple tissues during growth and development of *Arabidopsis*. Furthermore, cold stress can directly enhance the transcriptional activity of the *MsCBF4* promoter in tobacco leaves.

### Overexpression of MsCBF4 enhances Frost tolerance in Arabidopsis

To further elucidate the mechanism by which *MsCBF4* enhances frost tolerance, transgenic *Arabidopsis* lines that overexpressing *MsCBF4* were constructed. Following screening, three homozygous transgenic lines with high transcription levels (#27, #33, and #41) were identified. qRT-PCR revealed that the *MsCBF4* expression was significantly higher in the overexpression (OE) lines than in the wild-type (WT) lines (Fig. S2).

**Fig. 5:**
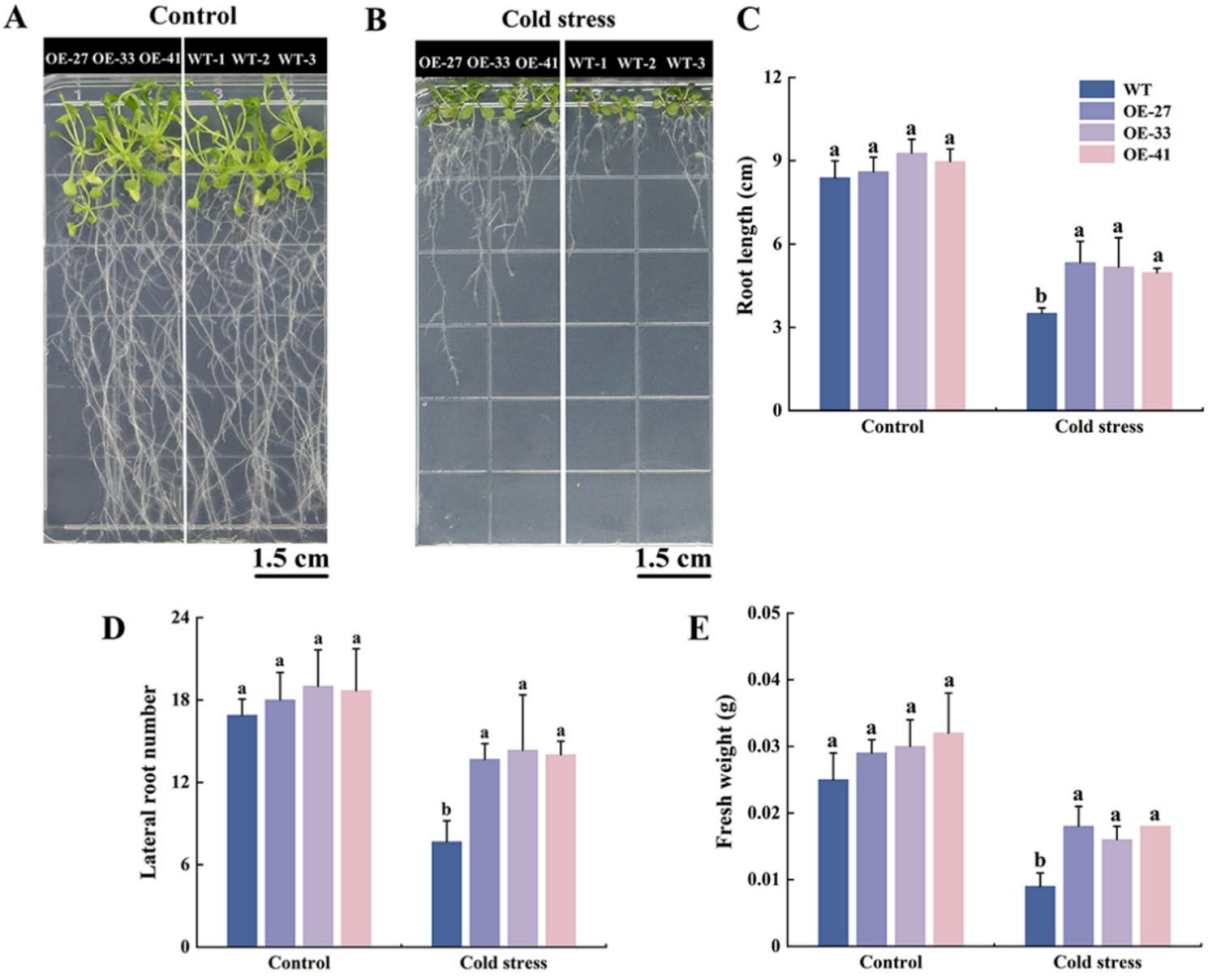
Phenotypic assay of *MsCBF4* transgenic *Arabidopsis* under cold stress. **A** 16-day-old *Arabidopsis* seedlings under normal growth condition. **B ***Arabidopsis* seedlings cultured under normal growth conditions for 6 days, followed by cold stress conditions (4℃) for 10 days. **C** Root length. **D** Number of lateral root. **E** Fresh weight. Data are presented as mean ± SD of three independent samples. Different letters indicate significant differences at the level of *Arabidopsis* lines (*P* < 0.05)

Different lines of *Arabidopsis* seedlings were subjected to cold treatment. Those seedlings grown under normal conditions for 16 days exhibited robust growth, showing no significant phenotypic differences (Fig. 5A). After 6 days under normal conditions, followed by 10 days at 4℃, all lines exhibited reduced growth compared to the control group. However, the OE lines displayed fewer wilted leaves and longer roots than the WT lines (Fig. 5B). Furthermore, no significant differences in root length, lateral root number and fresh weight were observed among the different lines in the control group. Following cold stress treatment, the OE lines exhibited significantly longer root length, greater number of lateral roots, and higher fresh weight compared to the WT lines (Fig. 5C-E). These results demonstrate that overexpression of *MsCBF4* enhances the growth-related traits of *Arabidopsis* under cold stress conditions.

**Fig. 6:**
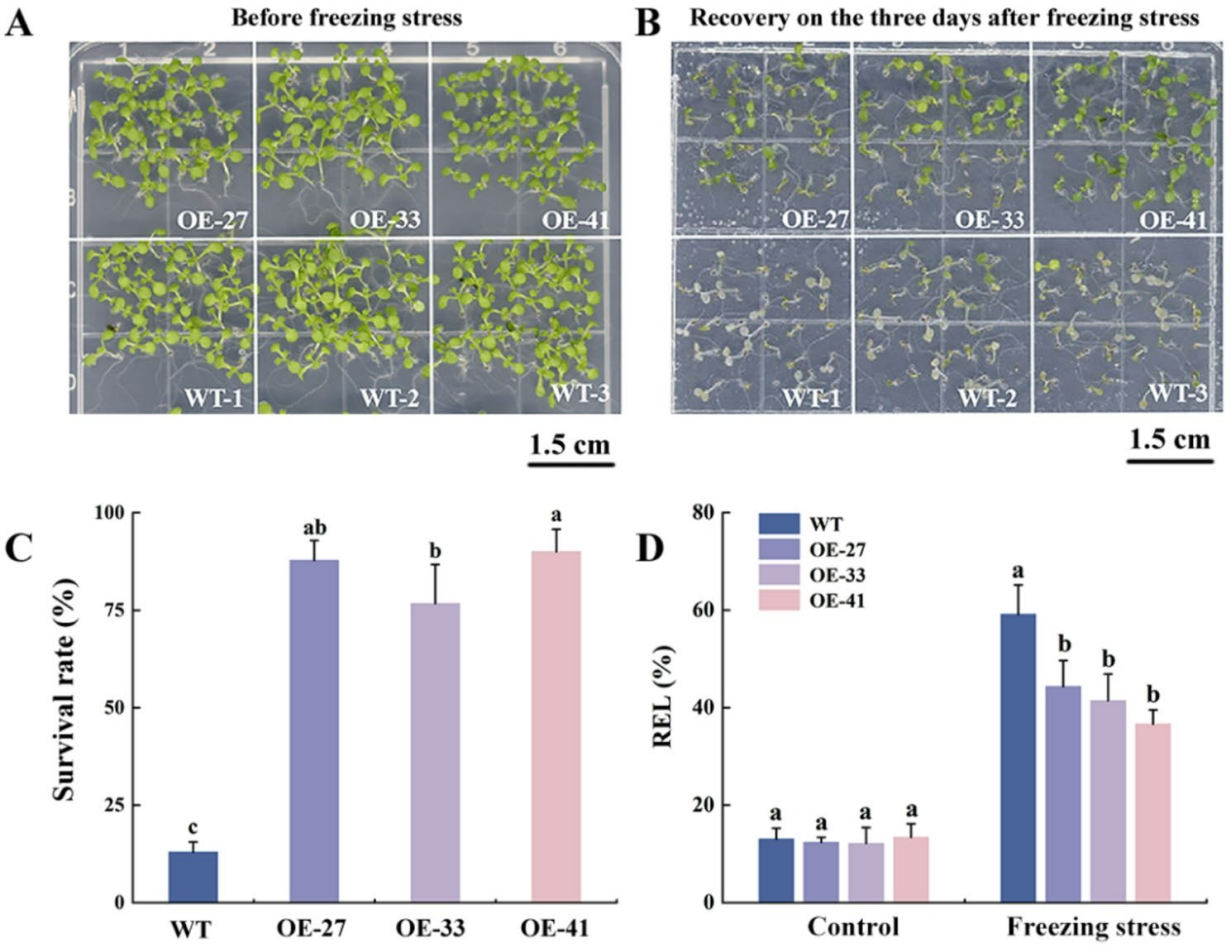
Effects of freezing stress treatment on *Arabidopsis* seedlings. **A** Phenotypes of OE and WT lines before freezing treatment. **B** Phenotypes after 3 h of freezing stress treatment at -5℃ and 3 days of recovery at room temperature. **C** Survival rates of each line after freezing stress treatment. **D** REL of each line before and after freezing stress exposure. Data are presented as mean ± SD of three independent samples. Different letters indicate significant differences at the level of *Arabidopsis* lines (*P* < 0.05)

*Arabidopsis* seedlings from different lines were subjected to freezing stress treatment at -5℃. Prior to treatment, the WT and OE lines exhibited consistent phenotypic characteristics (Fig. 6A). Following 3 days of recovery post-freezing treatment, the OE lines maintained most of their green pigmentation, whereas the WT lines exhibited complete wilting and widespread bleaching of tissues (Fig. 6B). Consistently, the survival rates of the three OE lines were significantly higher than that of the WT lines (Fig. 6C). Furthermore, the relative electrolyte leakage (REL) levels of each line were consistent across all lines prior to freezing stress exposure. Following freezing stress treatment, the REL of the OE lines was significantly lower than that of the WT lines (Fig. 6D). Collectively, these findings demonstrate that overexpression of *MsCBF4* significantly enhances the frost tolerance of *Arabidopsis* seedlings.

**Fig. 7:**
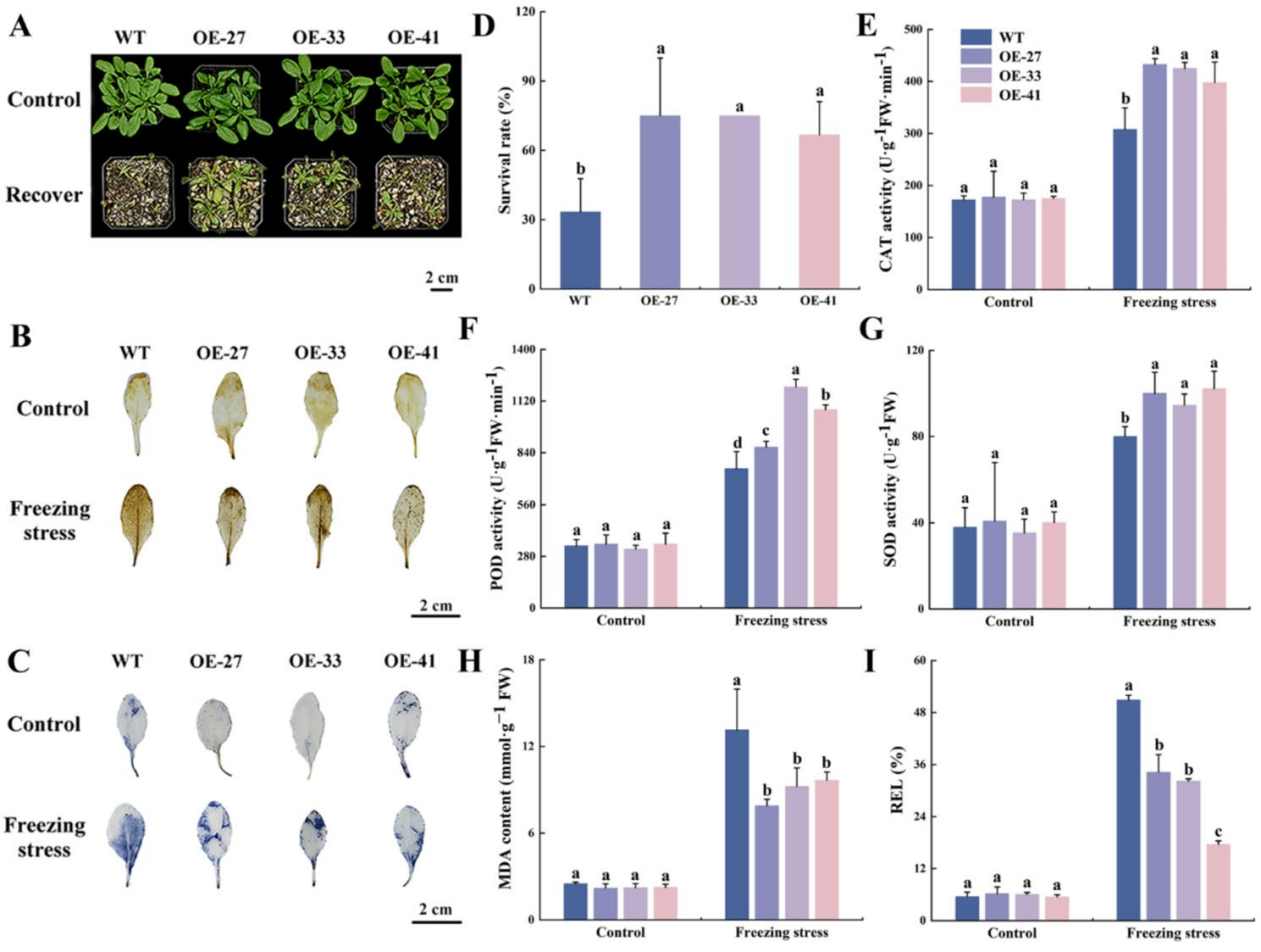
Effects of freezing treatment on mature *Arabidopsis.***A** Phenotypic change before and after 3 h of freezing treatment and 7 days of recovery at room temperature. **B** DAB staining to detect H₂O₂ levels in *Arabidopsis*. **C** NBT staining to detect O₂⁻ levels in *Arabidopsis*. **D** Survival rate. **E** CAT activity in *Arabidopsis*. **F** POD activity. **G** SOD activity. **H** MDA content. **I** REL. Data are presented as mean ± SD of three independent samples. Different letters indicate significant differences among different *Arabidopsis* lines (*P* < 0.05)

Subsequently, the freezing stress resistance of different *Arabidopsis* lines at the mature stage was evaluated. Under control conditions, no significant differences were observed between the OE and WT lines. Following freezing treatment at -5℃ for 3 h and 7 days of recovery under room temperature, the OE lines largely resumed normal growth, whereas the WT lines exhibited varying degrees of wilting and yellowing, with significantly reduced growth and survival compared to the OE lines (Fig. 7A, D). Histochemical staining with 3,3’-Diaminobenzidine (DAB) and nitro blue tetrazolium (NBT) revealed that, the OE lines showed weaker staining intensity than WT lines after freezing stress (Fig. 7B, C), indicating lower accumulation of H_2_O_2_ and O_2_^−^ in the OE lines. Further determination of abiotic stress-related physiological indicators revealed no significant differences among lines under control conditions. After freezing treatment, stress-related indicators changed in all lines, notably, the activities of catalase (CAT), peroxidase (POD), and superoxide dismutase (SOD) in the OE lines were significantly higher than those in the WT lines (Fig. 7E-G), while the accumulation of malondialdehyde (MDA) and REL was significantly lower in the OE lines (Fig. 7H, I). Collectively, these results demonstrate that overexpression of *MsCBF4* enhances frost tolerance in *Arabidopsis* by reducing ROSs accumulation, increasing antioxidant enzyme activity, and mitigating membrane damage.

### MsCBF4 induces the expression of abiotic stress-related genes in Arabidopsis

**Fig. 8:**
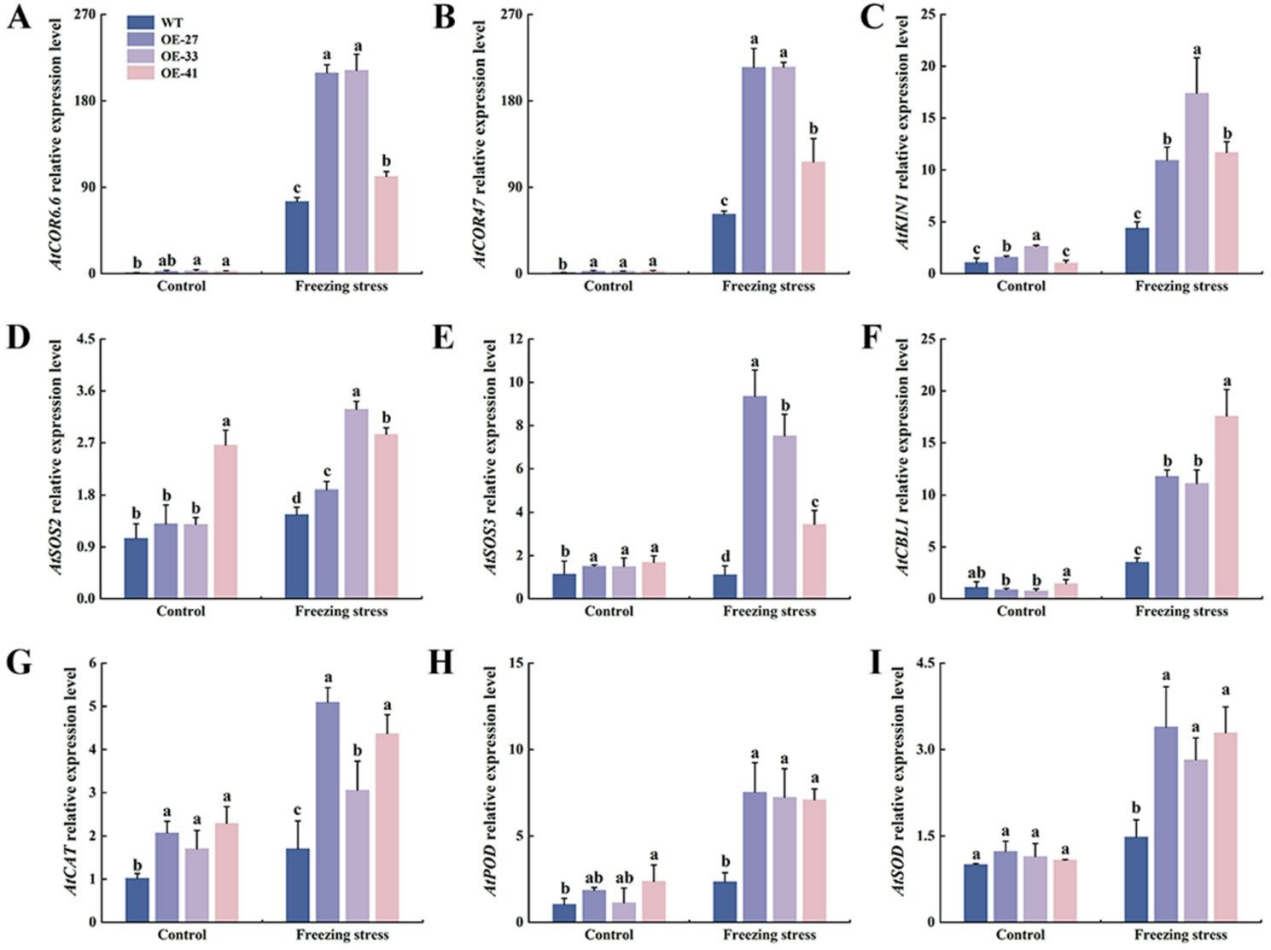
Expression of the abiotic stress-related genes in OE and WT lines under freezing stress. All *Arabidopsis* lines were cold-adapted at 4 °C for 8 h, followed by freezing at -5 °C for 3 h. **A ***AtCOR6.6*. **B ***AtCOR47*. **C ***AtKIN1*. **D ***AtSOS2*. **E ***AtSOS3*. **F ***AtCBL1*. **G ***AtCAT*. **H ***AtPOD*. **I ***AtSOD*. Data are presented as mean ± SD of three independent samples. Different letters indicate significant differences among different *Arabidopsis* lines (*P* < 0.05)

To investigate the regulatory mechanism of *MsCBF4* expression, expression levels of genes associated with abiotic stress, including those involved in responses to cold, drought, and salinity were analyzed. Under non-stress conditions, the expression levels of *AtCOR6.6/47*, *AtKIN1*, *AtSOS2/3*,* AtCBL1*, *AtCAT*,* AtPOD*, and *AtSOD* were comparable between OE and WT lines. After freezing treatment, these genes was significantly upregulated in both genotypes. Notably, their transcript levels in the OE lines were markedly higher than those in the WT lines (Fig. 8). These findings demonstrate that *MsCBF4* may be involved in regulating gene expression associated with abiotic stress and ROSs scavenging in *Arabidopsis*.

### *MsERF6* activates the expression of the *MsCBF4* promoter

**Fig. 9:**
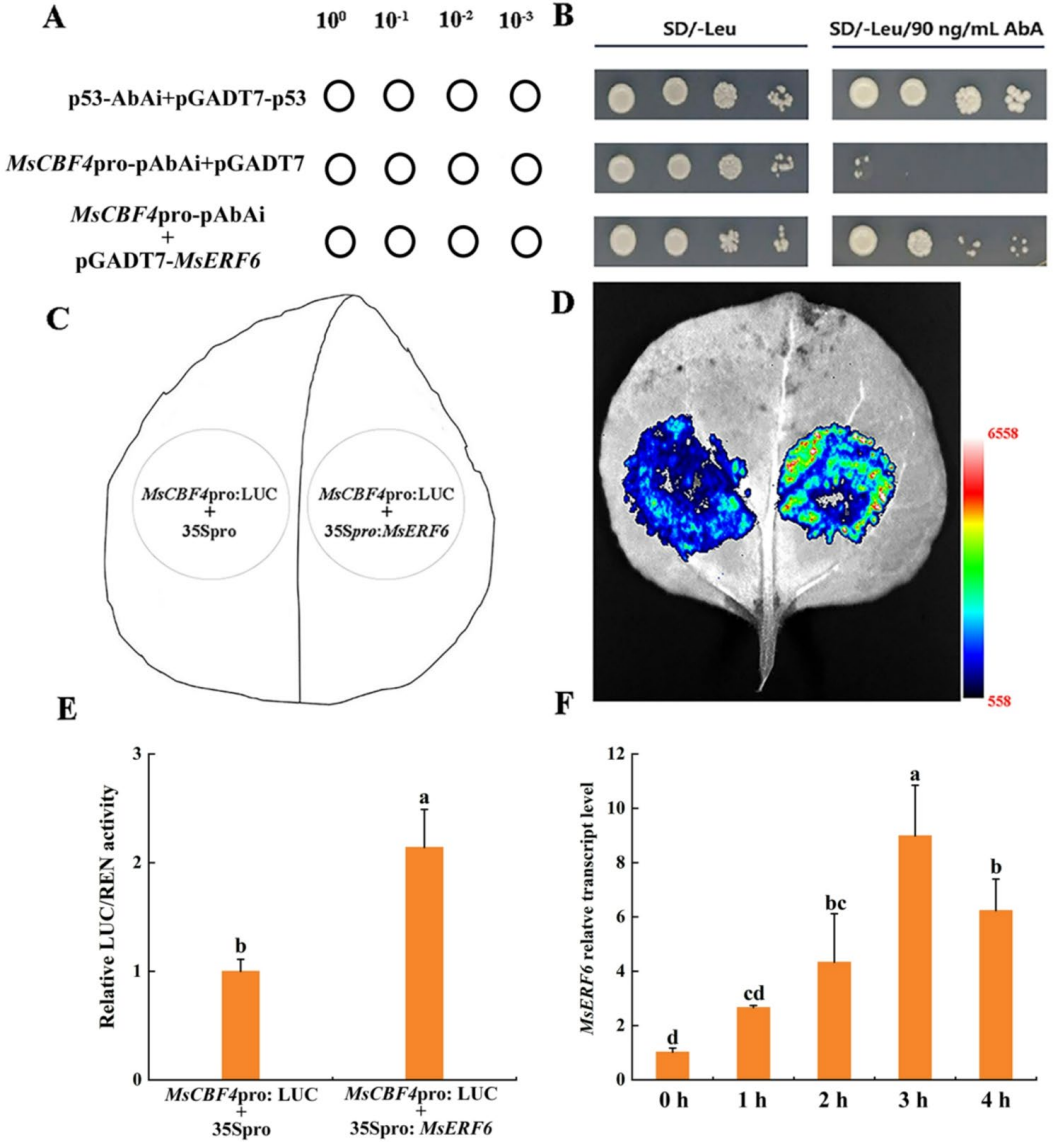
MsERF6 activates the *MsCBF4* promoter. **A** Schematic diagram of the Y1H assay. **B** Validation of the regulatory relationship between MsERF6 and the *MsCBF4* promoter using the Y1H assay. **C** Schematic diagram of the Dual-LUC assay. **D** Detection of the activating effect of MsERF6 on the *MsCBF4* promoter using the Dual-LUC assay. **E** Relative LUC/REN activity. **F** Dynamic expression of *MsERF6* in alfalfa leaves under freezing stress (-5℃). Data are presented as mean ± SD of three independent samples, and different letters indicate significant differences (*P* < 0.05)

To investigate the regulatory relationship between TFs and *MsCBF4* in alfalfa, an *MsCBF4*pro-pAbAi bait was conducted. Self-activation of the *MsCBF4*pro-pAbAi bait was effectively inhibited on SD/-Ura medium supplemented with 90 ng/mL Aureobasidin A (AbA) (Fig. S3). Screening of a Y1H cDNA library using the *MsCBF4*pro-pAbAi bait identified MsERF6 as a potential interacting prey protein, suggesting that MsERF6 may bind to the *MsCBF4* promoter. To further validate the regulatory interaction between MsERF6 and the *MsCBF4* promoter, Y1H assays were performed using the pGADT7-*MsERF6* prey vector and *MsCBF4*pro-pAbAi bait construct (Fig. 9A). All Y1H yeast transformants grew normally on SD/-Leu medium. In contrast, on SD/-Leu screening medium supplemented with 90 ng/mL AbA, only yeast cells co-transformed with pGADT7-*MsERF6* and *MsCBF4*pro-pAbAi exhibited normal growth, whereas the negative control showed no growth (Fig. 9B). These results provide direct evidence that MsERF6 positively regulates *MsCBF4* by binding to its promoter.

Furthermore, Dual-LUC assays were performed to measure luciferase activity in tobacco leaves (Fig. 9C). When *MsCBF4*pro: LUC and 35Spro: *MsERF6* were co-expressed, the fluorescence signal on tobacco leaves was significantly enhanced, and the relative LUC/sea cucumber luciferase (REN) activity was markedly increased compared to the control (Fig. 9D, E). These results provide evidence that MsERF6 can bind to the promoter region of *MsCBF4* in plants and activate its transcription. Further, the expression levels of *MsERF6* and *MsCBF4* were significantly upregulated under freezing stress (Fig. 9F). These results suggests that both *MsERF6* and *MsCBF4* are freeze-induced genes.

## Discussion

In plants, the AP2/ERF family is a large family of proteins which may be divided into four subfamilies: AP2, ERF, DREB, and RAV, as well as a few unclassified subgroups based on domain number and characteristic sequences. The AP2 subfamily is distinguished by the presence of two repeated AP2 domains, whereas the ERF, DREB, and RAV subfamilies each contain one. Members of the RAV subfamily are characterized by a single B3 domain [18, 19]. In this study, the AA sequence of *MsCBF4* contains only one AP2/ERF conserved domain comprising 58 AA. To further determine its subfamily classification, the AA sequence of this AP2 domain was compared with those of homologous genes from other species. The MsCBF4 protein clusters with known DREB/CBF proteins and is closely related to the MtCBF4 from *Medicago truncatula*. This evidence suggests that *MsCBF4* is best classified as a member of the DREB subfamily. The MsCBF4 protein is localized to the cell nucleus, which is consistent with the localisation of most AP2/ERF family TFs [13, 20, 21]. Full-length MsCBF4 protein and its C-terminal region strongly activate GAL4 reporter gene expression in yeast, whereas the truncated form lacking the C-terminal region loses the ability to activate GAL4 reporter gene completely. This is consistent with general AP2/ERF features. For example, *CbuERF114* in *Catalpa bungei* possesses a C-terminal activation domain [22], and the transcription activation activity of *TaAP2-10* in *Triticum aestivum* depends on its C-terminal region [23].

Analyzing the tissue expression patterns of target genes is important for understanding their function [24]. This study revealed that *MsCBF4* may exhibit differential expression across alfalfa tissues. CBF genes have been reported to play an important role in the plant’s responses to abiotic stress [11]. Changes in the *MsCBF4* expression in roots and leaves were examined during cold and freezing treatment. Cold stress was found to upregulate *MsCBF4*, suggesting that its production may be a response of plants to cold temperatures. Notably, *MsCBF4* expression is higher in leaves than in roots under freezing stress, whereas it is higher in roots than in leaves under cold stress. One possibility is that leaves directly sense the sudden temperature drop and form ice crystals under freezing stress, thereby promoting preferential *MsCBF4* expression. Under cold stress, roots continuously perceive low-temperature signals from the soil and coordinates adaptive responses, leading to high *MsCBF4* expression [2, 25]. This may explain the differential response of *MsCBF4* responses in roots and leaves under different stresses.

Previous studies have demonstrated that the overexpression of *PmhCBFc* from *Prunus mume* in *Arabidopsis* enhances cold tolerance and oxidative stress resistance [26]. Similarly, the overexpression of *StCBF1* and *StCBF4* from *Solanum tuberosum* conferred cold tolerance to *Arabidopsis* [27]. Likewise, overexpression of *JcCBF2* from *Jatropha curcas* improved frost tolerance in *Arabidopsis* during the initial stages of freezing stress [28]. The present results indicate that the overexpression of the alfalfa *MsCBF4* gene in *Arabidopsis* seedlings enhances cold tolerance, significantly increasing root length, lateral root number, and fresh weight under cold conditions. Overexpression of *MsCBF4* is also associated with improved frost tolerance of *Arabidopsis* seedlings, resulting in higher survival rates and reduced ion leakage compared to the WT lines. Consistent with these findings, the visual phenotype of mature *Arabidopsis* with and without the expression of *MsCBF4* after freezing stress supported this premise. Taken together, these lines of evidence suggest that overexpression of *MsCBF4* confers higher frost tolerance to *Arabidopsis* after freezing treatment.

ROSs are chemicals produced when plants are subjected to external stress, and excessive ROSs accumulation damages proteins, lipids, carbohydrates, DNA, and other components, leading to cell death [29]. H₂O₂ and O₂⁻ are examples of ROSs species which are produced during oxidative stress responses associated with cold stress in plants [30]. In this study, DAB and NBT staining revealed that freezing stress increased ROSs levels in mature *Arabidopsis* leaves, but ROSs levels in *MsCBF4*-overexpression lines were lower than those in the WT lines. When plants are subjected to external stress, ROSs are not the only molecules produced. Plants also produce a series of antioxidant protective enzymes are produced to clear ROSs and protect plant structures from damage caused by oxygen free radicals [31]. After freezing, CAT, POD, and SOD activities increased significantly increased in all lines, and were highest in the OE lines. Specifically, *AtCAT*, *AtPOD*, and *AtSOD* expression levels were significantly higher in the OE lines than in the WT lines after freezing. Overexpression of *MsCBF4* increased antioxidant enzyme levels in *Arabidopsis* and inhibited the excessive accumulation of ROSs. MDA content reflects the severity of membrane lipid peroxidation under adverse conditions, whereas REL is a key indicator of cell membrane integrity [32, 33]. In this study, no significant differences in MDA or REL were observed among the lines before freezing stress. After freezing, MDA accumulation in the OE lines was significantly lower than in the WT lines. Studies in *Vitis vinifera* show that overexpression of *VvERF63* improves cellular damage outcomes, increases antioxidant enzyme levels, and reduces ROSs levels [34]. In *Solanum lycopersicum*, antisense *SlACS2* fruits exhibit severe cold damage, characterized by reduced antioxidant enzyme activity, elevated MDA content, and increased ion leakage [35]. These findings indicate that *MsCBF4* may likewise effectively enhance frost tolerance in *Arabidopsis*. However, its specific function within the alfalfa genetic background may differ due to its complex interactions with endogenous regulatory networks. Future studies will precisely elucidate its cold tolerance function through overexpression or knockout experiments of the *MsCBF4* gene in alfalfa.

As a TF, CBF can recognize CRT/DRE cis-acting elements in the promoters of downstream target genes, initiating transcription [36]. In *Dimocarpus longan*, *DlCBF1/2/3* induces *AtRD29A*, *AtCOR15A*, *AtCOR47*, and *AtKIN1* by binding to CRT/DRE cis-acting elements, thereby promoting cold adaptation process in *Arabidopsis* [37]. In addition, *COR6.6* and *COR15B* are also affected by cold-stress responses [38, 39]. *AtCBL1*, *AtSOS2*, and *AtSOS3* participate in the SOS signaling pathway of salt stress [40]. Furthermore, the *SoSOS1* in *Saccharum officinarum* is significantly upregulated under cold stress [41]. Responses of *OsCBL1* and *OsCBL3* in *Oryza sativa* to cold stress have also been reported [42]. These abiotic stress-related target genes were analyzed by qRT-PCR and were found to be upregulated by freezing stress in each line, with significantly higher expression in OE plants than in WT lines. This suggests that *MsCBF4* may activate the CBF-COR pathway and enhance tolerance indirectly by coordinating with SOS signalling pathways.

Analyzing the function of promoters is key to exploring the transcriptional regulation mechanism of TFs [43]. This study revealed the expression patterns of the *MsCBF4* promoter in different tissues during the vegetative growth stage of *Arabidopsis* using GUS staining. The *MsCBF4* promoter was found to be active in *Arabidopsis* seedlings, mature rosette leaves, inflorescences, and pods, indicating its presence in various tissues. In addition, transient expression experiments showed that the *MsCBF4* promoter can be directly induced by cold. Previous studies have reported that *SlERF15* in *Solanum lycopersicum* activates *SlCBF1* transcription by binding to its promoter [44], and that *VviERF11-c* in *Vitis vinifera* specifically binds to the *VvCBF* promoter [45]. Similarly, Y1H and Dual-LUC assay provided evidence that MsERF6 can bind to the *MsCBF4* promoter and positively regulate *MsCBF4* expression. Sequence analysis further revealed an ethylene-response element, ERE (ATTTTAAA), in the *MsCBF4* promoter. Since ERF and DREB proteins can bind to DRE/CRT or ERE elements [46], MsERF6 protein may interact with *MsCBF4* by binding to the ERE element. Futhermore, spatio-temporal expression analysis showed that *MsERF6* expression was gradually upregulated with prolonged freezing treatment. Our study suggests that MsERF6 specifically binds to the *MsCBF4* promoter and positively regulates its expression. The transcription level of *MsERF6* is also induced by freezing environments. However, the downstream gene network regulated by *MsCBF4* remains unclear. In subsequent studies, DNA affinity purification sequencing (DAP-Seq) technology will be employed to identify the downstream target genes of *MsCBF4*, thereby clarifying the signalling pathway underlying *MsCBF4*-mediated freezing stress responses.

## Conclusions

In summary, *MsCBF4* is a TF cloned from alfalfa that encodes a nuclear-localized protein with transcriptional activation activity, and its C-terminal region serves as the activation domain. *MsCBF4* is most highly expressed in roots and its expression is markedly upregulated in both roots and leaves under cold and freezing stress. In transgenic *Arabidopsis*, *MsCBF4* significantly increases root length, number of lateral roots, and fresh weight under cold stress. Furthermore, *MsCBF4* enhances frost tolerance by reducing ROSs accumulation, increasing antioxidant enzyme activity, and mitigating membrane damage. *MsCBF4* participates in the CBF-COR and SOS signaling pathways, which collectively contribute to improved freezing tolerance of transgenic *Arabidopsis*. The *MsCBF4* promoter exhibits transcriptional activity in response to cold and is regulated by MsERF6 through direct binding to the promoter. Taken together, these findings suggest that *MsCBF4* could enhance frost tolerance in *Arabidopsis*.

## Methods

### Alfalfa cultivation and freezing treatment

The alfalfa seeds of variety “Dongnong NO.1” used in this study were provided by the Grassland Science Laboratory of Northeast Agricultural University. To break seed dormancy in the alfalfa genotype “Dongnong NO.1”, seeds were incubated at 50℃ for 2 h. They were then transferred to pots containing a 2:1 mixture of nutrient soil and vermiculite and grown in a controlled environment at 25℃ with a 16-hour light/8-hour dark photoperiod. At 13 weeks, healthy seedlings with uniform growth were selected, and tissues were collected from roots, stems, leaves, flowers, and pods.

Cold stress treatments were applied at the 5-week growth stage. For cold treatment, plants were exposed to 4℃ for 0, 1, 3, 6, 12, and 24 h. For freezing treatment, plants were first adapted at 4℃ for 2 days, after which the temperature was gradually lowered to − 5℃ at a rate of 3℃ per hour. Plants were then subjected to freezing for 0, 1, 2, 3, and 4 h. Root and leaf tissues were collected separately. Each treatment included three biological replicates. All samples were immediately frozen in liquid nitrogen after collection and stored at − 80℃ until gene expression analysis.

### Gene cloning and sequence analysis

Total RNA was extracted from leaves of “Dongnong NO.1” using the RNAprep Pure Plant Kit (TianGen, Beijing, China) following the manufacturer’s instructions. cDNA was synthesized using HiScript^®^ III RT SuperMix (Vazyme, Nanjing, China). Genomic DNA was extracted using Fast Plant Genomic DNA Extract (Coolaber, Beijing, China). Specific primers were designed based on the CDS and upstream flanking regulatory region sequence of *MS.gene006341.t1* in the genome of alfalfa genotype “XinjiangDaye” (Table S3). The target sequence was amplified using cDNA and DNA as templates. Sequences were aligned using DNAMAN (Version 6.0) and a phylogenetic tree constructed using MEGA (Version 7.0).

Bioinformatics predictions were performed using the following tools: SignalP 4.0 (http://www.cbs.dtu.dk/services/SignalP/, accessed May 12, 2024), TMHMM Server 2.0 (http://www.cbs.dtu.dk/services/TMHMM/, accessed May 12, 2024), ExPASy PROSITE (https://prosite.expasy.org/, accessed May 10, 2024), CELLO (http://cello.life.nctu.edu.tw/, accessed May 12, 2024), SOPMA (https://npsa-prabi.ibcp.fr/, accessed May 15, 2024), and PlantCARE (http://bioinformatics.psb.ugent.be/, accessed May 15, 2024).

### Subcellular localization of MsCBF4 protein

Seeds of the tobacco “*Nicotiana benthamiana*” were provided by the Crop Stress Molecular Biology Lab at Heilongjiang Bayi Agricultural University. The pCAMBIA1302-GFP vector was used as the plant expression vector. The *MsCBF4* coding sequence, lacking a stop codon, was fused in-frame with mGFP5 at the 5′ end to enable expression [47]. The recombinant plasmid *MsCBF4*-GFP and the marker *AHL22-*mRFP were separately introduced into *A. tumefaciens* GV3101 (p19 + psoup) via the heat-shock method. These two *Agrobacterium* strains were co-infiltrated into tobacco leaves, and after 3 days of transient expression, subcellular localization of *MsCBF4*-GFP was examined using laser confocal microscopy (Zeiss LSM 800, Germany). Excitation wavelengths were 488 nm for GFP and 587 nm for mRFP, with emission detected at 400–580 nm (GFP) and 580–620 nm (mRFP).

### Verification of yeast cell MsCBF4 transcription activation

The coding sequences of the full-length *MsCBF4* (0–543 bp), N-terminal (0–51 bp), AP2-terminal (52–225 bp), and C-terminal (226–543 bp) regions were cloned into the pGBKT7 vector. Then, the constructed vector was transformed into Y2H yeast cells. The cells were cultured at 30℃ for 3 days on SD/-Trp, SD/-Trp/-His, and SD/-Trp/-His/-Ade (X-α-gal) media, and transcription activation characteristics were observed.

### Obtaining transgenic Arabidopsis plants and freezing stress treatment

Seeds of *Arabidopsis* “Columbia ecotype” were provided by the Crop Stress Molecular Biology Lab at Heilongjiang Bayi Agricultural University. *Arabidopsis* seeds were disinfected and placed on solidified Murashige and Skoog (MS) medium (containing 3% sucrose, pH 5.8), followed by a vernalization treatment at 4℃ for 2 days. After 7 days of cultivation at room temperature, the seedlings were transplanted into a mixture of peat soil, perlite, and vermiculite (1:1:1) and cultivated in a growth chamber at 22℃ under a 16/8 h (light/dark) cycle for 6 weeks. The *A. tumefaciens* GV3101, containing the plant expression vector *MsCBF4*-GFP, was employed to infect the inflorescences of *Arabidopsis* [48]. The first batch of seeds was designated as the T_0_ generation. These seeds were collected and disinfected, then sown on solidified MS medium. After disinfection, T_0_ seeds were germinated on solidified MS medium, and T_1_ seedlings were selected on medium containing 25 µg/mL hygromycin. This selection process was repeated until T_3_ homozygous lines were obtained.

WT plants and overexpressing T_3_ lines (OE-27, 33, and 41) were used for stress treatments. For cold tolerance experiment, after 6 days of cultivation on solidified MS medium, three uniformly developed seedlings per line were selected and transferred to new medium. They were then maintained under normal conditions and cold conditions (4℃) for 10 days, and their root length, lateral root number, and fresh weight were measured. For the freezing experiment, 30 seeds were sown on solidified MS medium, vernalized, and cultured at room temperature for 5 days. During the seedling stage, plants were cold-adapted at 4℃ for 3 days, after which the temperature was gradually reduced to − 5℃ at a rate of 3℃ per hour. Leaf tissue was collected after 3 h of freezing treatment. To induce freezing stress in mature *Arabidopsis*, germinated seedlings were transplanted into soil (four plants per pot) and grown for 3 weeks. Plants were then cold-adapted at 4℃ for 8 h, followed by a gradual decrease in temperature to − 5℃ (3℃ per hour). Leaf tissue was harvested after 3 h of freezing treatment. All treatments were performed in triplicate. Collected samples were immediately frozen in liquid nitrogen and stored at − 80℃ until further analysis.

### Measurement of physiological and biochemical indicators

Production of O₂⁻ was detected using the NBT method, and the production of H₂O₂ was detected using the DAB method [49]. Activities of antioxidant enzymes CAT, POD, and SOD, as well as MDA content, were analyzed following existing methodology [50]. Determination of REL was conducted using the conductivity method [51]. Each experiment was carried out three times.

### GUS staining

The pCAMBIA3301-GUS vector was used as the plant expression vector, with the native CaMV 35 S promoter replaced by the *MsCBF4* promoter and fused to the GUS reporter gene at the 5′ end to enable expression [52]. *Arabidopsis* plants were transformed using *A. tumefaciens* GV3101, and T_1_ transgenic lines were selected on solidified MS medium containing 25 µg/mL glufosinate. Screening was continued until stable T_3_ lines were obtained.

For tissue-specific expression analysis, samples were collected from 1-week-old seedlings, 7-week-old rosette leaves, inflorescences, and pods, with three biological replicates for each tissue type. GUS staining was performed using the GUS Stain Kit (Coolaber, Beijing, China) for 6 h in the dark. After chlorophyll removal, tissues were observed under a stereomicroscope (Leica EZ4 W, Germany).

### Analysis of MsCBF4 promoter activity

The promoter of *MsCBF4* was ligated into the pGreen II 0800-LUC vector. Transform the fusion expression vector *MsCBF4*pro: LUC into *A. tumefaciens* GV3101 (p19 + psoup). Subsequently, transient gene expression was performed in tobacco leaves. After 48 h of cultivation at room temperature under low light conditions, plants were further cultured for 3 h under either room temperature or cold stress (4℃). Luminescence was visualized using a chemiluminescence imaging system (Tanon 5200, China). LUC activities were quantified using the Dual Luciferase Reporter Assay Kit (Vazyme, Nanjing, China). Three biological replicates per experiment were carried out.

### Y1H assay

The promoter sequence of *MsCBF4* was inserted into the pAbAi vector as a bait, and the recombinant plasmid *MsCBF4*pro-pAbAi was transformed into Y1H Gold yeast cells using the LiAC-mediated competent cell preparation method to prepare competent cells [53]. The “Dongnong NO.1” nuclear system yeast library plasmid was then transformed into the prepared Y1H Gold competent cells so that upstream genes may be screened from the product. The *MsERF6* CDS was inserted into the pGADT7 vector to construct the prey, which was then transformed into the prepared Y1H Gold competent cells. The cells were cultured at 30℃ for 3 days in SD/-Leu medium with or without AbA.

### Dual-LUC assay

The *MsCBF4* promoter was cloned into the pGreen II 0800-LUC vector to generate the reporter construct, while the *MsERF6* CDS was inserted into the pGreen II 62-SK vector to serve as the effector [54]. Both constructs were separately transformed into *A. tumefaciens* GV3101 (p19 + psoup) and co-infiltrated into tobacco leaves, with three biological replicates for each combination. After co-culturing for 3 days under room temperature and low light conditions, the chemiluminescence imaging system and Dual Luciferase Reporter Assay Kit were used to measure the LUC and REN activities.

### qRT-PCR and statistical analysis of data

qRT-PCR detection was performed following established methodology [55], with specific primers listed in Table S3. The CFX 96 Real-Time PCR Detection System (Bio-Rad, USA) was used. Relative abundance values were normalized to the internal reference gene, and the relative expression levels of the target genes were calculated using the 2^−ΔΔCt^ method [56]. Each experiment included three biological replicates, with each biological replicate comprising three technical replicates. All data were analyzed using one-way analysis of variance (ANOVA) and Student’s *t*-test with SPSS software (SPSS Inc., Chicago, IL, USA, version 19.0), and differences were tested for significance using Duncan’s multiple range test (*P* < 0.05). Data visualization and analysis were performed using Origin 2018 64-bit software.

## Supplementary Information

Additional file 1: Supplementary Figure S1. Prediction of the tertiary structure of the amino acid sequence of MsCBF4 and its homologous proteins. A MsCBF4. B MtCBF4. C CaDREB1A. D GmDREB7. E CsDREB1A. F NtDREB1A. G AtCBF3. H GsDREB1F. I TaDREB1B. J OsDREB1E. Supplementary Figure S2. Identification of MsCBF4 transgenic Arabidopsis. A Hygromycin screening of seven-day-old plants of the WT and MsCBF4 transgenic lines. B Agarose gel electrophoresis detection and C qRT-PCR detection. Data are presented as mean ± SD of three independent samples, and different letters indicate significant differences (P < 0.05). Supplementary Figure S3. MsCBF4pro-pAbAi self-activation inhibition of Aureobasidin A (AbA) concentration screening. Supplementary Table S1. RNA-Seq results of the MS.gene006341.t1 gene in alfalfa “Dongnong NO.1”. Supplementary Table S2. Sequence analysis of MsCBF4. Supplementary Table S3. Primers used in this study.

## Data Availability

The sequence data of *MsCBF4* can be obtained from the NCBI database with accession number: PRJNA1217929 (https://www.ncbi.nlm.nih.gov/sra/). All analyzed or generated data is included in this article. The data analyzed or generated in this study can be obtained from the corre sponding author with upon reasonable request.
