# Supplementary material for: The alfalfa AP2/ERF transcription factor MsCBF4 enhances frost tolerance in Arabidopsis thaliana
Source: BMC Plant Biol. 2025 Nov 24;25:1763. doi: 10.1186/s12870-025-07778-y (PMC12752023; doi:10.1186/s12870-025-07778-y)
Supplement: Supplementary file 1 — Additional file 1: Supplementary Figure S1. Prediction of the tertiary structure of the amino acid sequence of MsCBF4 and its homologous proteins. A MsCBF4. B MtCBF4. C CaDREB1A. D GmDREB7. E CsDREB1A. F NtDREB1A. G AtCBF3. H GsDREB1F. I TaDREB1B. J OsDREB1E. Supplementary Figure S2. Identification of MsCBF4 transgenic Arabidopsis. A Hygromycin screening of seven-day-old plants of the WT and MsCBF4 transgenic lines. B Agarose gel electrophoresis detection and C qRT-PCR detection. Data are presented as mean ± SD of three independent samples, and different letters indicate significant differences (P < 0.05). Supplementary Figure S3. MsCBF4pro-pAbAi self-activation inhibition of Aureobasidin A (AbA) concentration screening. Supplementary Table S1. RNA-Seq results of the MS.gene006341.t1 gene in alfalfa “Dongnong NO.1”. Supplementary Table S2. Sequence analysis of MsCBF4. Supplementary Table S3. Primers used in this study. [file 12870_2025_7778_MOESM1_ESM.zip › Supplementary material/Figure S2.pdf]

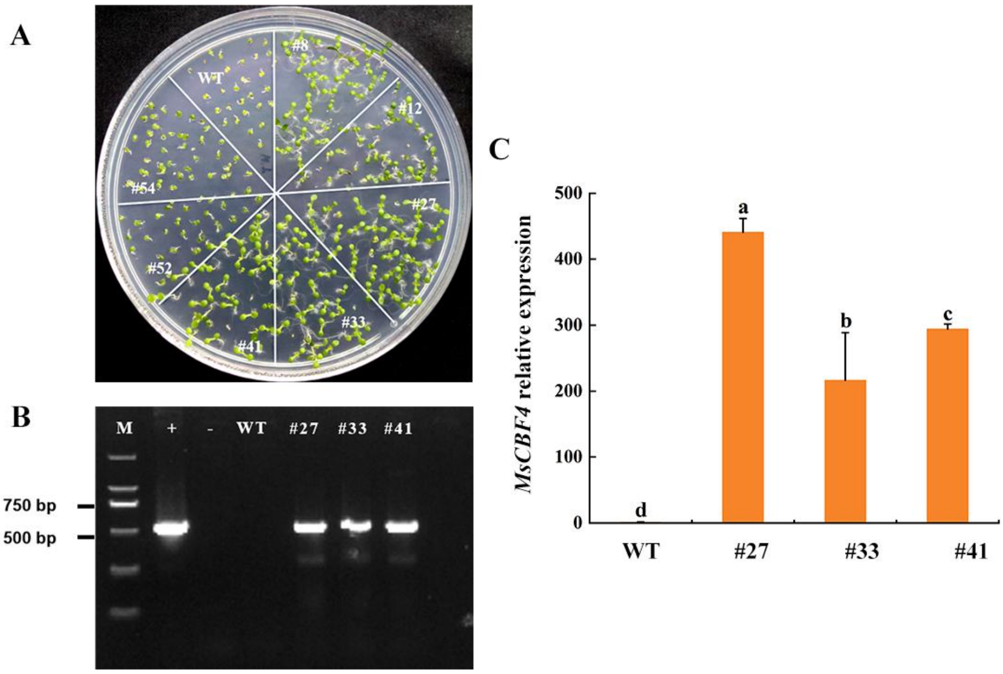

Fig. S2 Identification of *MsCBF4* transgenic *Arabidopsis*. **A** Hygromycin screening of seven-day-old plants of the WT and *MsCBF4* transgenic lines. **B** Agarose gel electrophoresis detection and **C** qRT-PCR detection. Data are presented as mean ± SD of three independent samples, and different letters indicate significant differences ($P < 0.05$).
