# Supplementary material for: The alfalfa AP2/ERF transcription factor MsCBF4 enhances frost tolerance in Arabidopsis thaliana
Source: BMC Plant Biol. 2025 Nov 24;25:1763. doi: 10.1186/s12870-025-07778-y (PMC12752023; doi:10.1186/s12870-025-07778-y)
Supplement: Supplementary file 1 — Additional file 1: Supplementary Figure S1. Prediction of the tertiary structure of the amino acid sequence of MsCBF4 and its homologous proteins. A MsCBF4. B MtCBF4. C CaDREB1A. D GmDREB7. E CsDREB1A. F NtDREB1A. G AtCBF3. H GsDREB1F. I TaDREB1B. J OsDREB1E. Supplementary Figure S2. Identification of MsCBF4 transgenic Arabidopsis. A Hygromycin screening of seven-day-old plants of the WT and MsCBF4 transgenic lines. B Agarose gel electrophoresis detection and C qRT-PCR detection. Data are presented as mean ± SD of three independent samples, and different letters indicate significant differences (P < 0.05). Supplementary Figure S3. MsCBF4pro-pAbAi self-activation inhibition of Aureobasidin A (AbA) concentration screening. Supplementary Table S1. RNA-Seq results of the MS.gene006341.t1 gene in alfalfa “Dongnong NO.1”. Supplementary Table S2. Sequence analysis of MsCBF4. Supplementary Table S3. Primers used in this study. [file 12870_2025_7778_MOESM1_ESM.zip › Supplementary material/Table S2.pdf]

Table S2 Sequence analysis of *MsCBF4*.

Gene name	Number of amino acids	Relative molecular mass	Theoretical isoelectric point	Instability index	Hydrophobicity	Transmembrane domain	Signal peptide	Subcellular localization prediction
MsCBF4	180	20443.92	5.76	64.25	Hydrophilic protein	None	None	Chloroplasts, cell nuclei, mitochondria
