# Supplementary material for: The alfalfa AP2/ERF transcription factor MsCBF4 enhances frost tolerance in Arabidopsis thaliana
Source: BMC Plant Biol. 2025 Nov 24;25:1763. doi: 10.1186/s12870-025-07778-y (PMC12752023; doi:10.1186/s12870-025-07778-y)
Supplement: Supplementary file 1 — Additional file 1: Supplementary Figure S1. Prediction of the tertiary structure of the amino acid sequence of MsCBF4 and its homologous proteins. A MsCBF4. B MtCBF4. C CaDREB1A. D GmDREB7. E CsDREB1A. F NtDREB1A. G AtCBF3. H GsDREB1F. I TaDREB1B. J OsDREB1E. Supplementary Figure S2. Identification of MsCBF4 transgenic Arabidopsis. A Hygromycin screening of seven-day-old plants of the WT and MsCBF4 transgenic lines. B Agarose gel electrophoresis detection and C qRT-PCR detection. Data are presented as mean ± SD of three independent samples, and different letters indicate significant differences (P < 0.05). Supplementary Figure S3. MsCBF4pro-pAbAi self-activation inhibition of Aureobasidin A (AbA) concentration screening. Supplementary Table S1. RNA-Seq results of the MS.gene006341.t1 gene in alfalfa “Dongnong NO.1”. Supplementary Table S2. Sequence analysis of MsCBF4. Supplementary Table S3. Primers used in this study. [file 12870_2025_7778_MOESM1_ESM.zip › Supplementary material/Table S3.pdf]

Table S3 Primers used in this study.

Primer	Sequence(5'-3')
KzMsCBF4-F	ATGCCGAAGAAGCGTGCAGG
KzMsCBF4-R	CTAAAATGAATAACTCCACAATGAAAT
1302MsCBF4-F	GGACTCTTGACCATGGTAATGCCGAAGAAGCGTGC
1302MsCBF4-R	CTTCTCCTTTACTAGTAAATGAATAACTCCACAATGAAATTTCAGTTTCATCA
BDMsCBF4-F	AGGCCGAATTCCCGGGGATCCATGCCGAAGAAGCGTGCA
BDMsCBF4-R	ATGCGGCCGCTGCAGGTCGACCTAAAATGAATAACTCCACAATGAAATTT
BDMsCBF4(0)-F	AGGCCGAATTCCCGGGGATCCATGCCGAAGAAGCGTGCA
BDMsCBF4(0)-R	ATGCGGCCGCTGCAGGTCGACAGGGTGACGTGTTTCTCTGAACT
BDMsCBF4(52)-F	AGGCCGAATTCCCGGGGATCCGTGTACCGTGGTGTAAGAAAGAGG
BDMsCBF4(52)-R	ATGCGGCCGCTGCAGGTCGACAGCAAAGTTAAGACAAGCAGACCTT
BDMsCBF4(226)-F	AGGCCGAATTCCCGGGGATCCGATTCTGCATGGAGGTTGCC
BDMsCBF4(226)-R	ATGCGGCCGCTGCAGGTCGACCTAAAATGAATAACTCCACAATGAAATTT
MsActin-F	CAAAAGATGGCAGATGCTGAGGAT
MsActin-R	CATGACACCAGTATGACGAGGTCG
QMsCBF4-F	GGGAAGTGGGTTTGTGAAGTAAGAG
QMsCBF4-R	ACGCAATCGCCGCAACATC
35S-F	ACGGGGGACTCTTGACCATG
QAtActin2-F	TGTGCCAATCTACGAGGGTTT
QAtActin2-R	TTTCCCGCTCTGCTGTTGT
QAtCBL1-F	GAAATGAAACTGGCTGATGAAACCATAGAG
QAtCBL1-R	CTCGTGGCAATCTACTCGGTCTTAAACC
QAtSOS2-F	ATTGAGGCTGTAGCGAAC
QAtSOS2-R	GGTATTCCTTCTGTTGCC
QAtSOS3-F	GGAGGAATCTCTTCGCTG
QAtSOS3-R	CACGAAAGCCTTATCCACC
QAtCOR6.6-F	GTCAGAGACCAACAAGAATGCC
QAtCOR6.6-R	TGACTCGAATCGCTACTTGTTC
QAtKIN1-F	TCAGAGACCAACAAGAATG
QAtKIN1-R	TTGTCCAGCAGAACATTG
QAtCOR47-F	ACCAACAAGAATGCCTTCCA
QAtCOR47-R	GCCGCATCCGATACACTCTTT
QAtCAT-F	GATGATAAGCTACTCCAGACCC
QAtCAT-R	TTGTTGTGGTGAGCACATTTAG
QAtPOD-F	CGAAAAGGACTCAACACAAGAG
QAtPOD-R	CGAGTTATCGTAAAGCCTACCT
QAtSOD-F	AGGAAACATCACTGTTGGAGAT
QAtSOD-R	GAGTTTGGTCCAGTAAGAGGAA
KzpMsCBF4-F	ATAAAATAATATGGAAATATTGTTTTTGTGGAAGGG
KzpMsCBF4-R	GGTTTCTGCTAGAAACCTCTCTG
3301MsCBF4-F	GAGCTCGGTACCCGGGGATCCATAAAATAATATGGAAATATTGTTTTTGTG
3301MsCBF4-R	TTACCCTCAGATCTACCATGGATAGTTTGGTGGAGCTGTTGCA
AbAiMsCBF4-F	GAAAAGCTTGAATTCGAGCTCATAAAATAATATGGAAATATTGTTTTTGTG

AbAiMsCBF4-R	GTCGACAGATCCCCGGGTACCATAGTTTGGTGGAGCTGTTGCA
KzMsERF6-F	ATGGCTAATTTTGAAGAAGTTTCAGC
KzMsERF6-R	TCACACAACAACTAAAGGTGACAAA
ADMsERF6-F	GTGGGCATCGATACGGGATCCATGGCTAATTTTGAAGAAGTTTCAGC
ADMsERF6-R	ATTCATCTGCAGCTCGAGCTCTCACACAACAACTAAAGGTGACAAAC
0800pMsCBF4-F	TTCCTGCAGCCCGGGGGATCCGACATGGATTAGTAATCCACTCGTTATT
0800pMsCBF4-R	GGCGGCCGCTCTAGAACTAGTATAGTTTGGTGGAGCTGTTGCA
62SKMsERF6-F	GGCGGCCGCTCTAGAACTAGTATGGCTAATTTTGAAGAAGTTTCAGC
62SKMsERF6-R	TTCCTGCAGCCCGGGGGATCCTCACACAACAACTAAAGGTGACAAAC
QMsERF6-F	AGCCGGAAGTTGCTGAGAAA
QMsERF6-R	GCCTGAAAGCAGCTGAATCG
